# Rare location of head and neck adenoid cystic carcinoma

**DOI:** 10.11604/pamj.2019.34.33.19245

**Published:** 2019-09-16

**Authors:** Asmaa Naim, Amal Hajjij, Faycal Abbad, Amal Rami, Mustapha Essaadi

**Affiliations:** 1 Casablanca Cancer Center, Hospital international Cheikh Khalifa, Casablanca, Morocco; 2 Head and Neck Department, Hospital International Cheikh Khalifa, Casablanca, Morocco; 3 Radiology Department, Hospital International Cheikh Khalifa, Casablanca, Morocco

**Keywords:** Adenoid cystic carcinoma, laryngeal tumor, minor salivary gland cancer

## Abstract

Adenoid Cystic Carcinoma of larynx is extremely rare location. We herein describe an unusual clinical and radiological presentation of ACCL and review recent literature. We report a case of a 38-year-old woman with history of asthma, presented to our department with acute inspiratory dyspnea that required an emergency tracheotomy. Physical examination revealed a large anterior cervical mass without any lymphadenopathy suspecting thyroid origin. Cervical Computed Scan showed a tumor process between the thyroid lobe, the left edge of the subglottic area and first tracheal rings filling all the lumen, discussing either a laryngo-tracheal or thyroid origin. The patient underwent a panendoscopy under general anesthesia that confirmed a subglottic extension of the tumor and multiples biopsies showed a malignant salivary origin of the mass. After multidisciplinary discussion, the patient underwent total laryngectomy and thyroidectomy with bilateral selective neck dissections (levels II- IV). Anatomopathological examination confirmed the laryngeal location of Adenoid Cystic Carcinoma classified pT4aN0R0. Adjuvant radiation therapy was indicated. In our knowledge, only 10 cases were reported in the literature with this unusual presentation. Moreover, the case we report is in the subglottic floor without invasion of neither vocal cords nor trachea. Total laryngectomy with neck dissection remains the recommended therapeutic procedure for locally advanced ACCL. Adverse features such as close or positive margins, T3-4, intermediate or high grade neural and perineural spread, lymphatic or vascular invasion or lymph node metastases should indicate adjuvant treatment to improve the outcome. The lack of randomized multicentric study, implies the management of ACCL by skilled multidisciplinary team, to suggest adequate personalized treatment.

## Introduction

Adenoid Cystic Carcinoma is a common tumor of salivary glands. Due to the very low density of minor glands in the larynx (23 - 47 glands/cm^2^) versus oral cavity for example (600-1000 glands/cm^2^), the incidence of adenoid cystic carcinoma of the larynx “ACCL” is extremely rare (<1% of all laryngeal cancer) [1]. Clinical symptomatology of ACCL is dominated by dyspnea and hoarseness. Subglottic location is clearly the most common while distribution by gender remains controversial [2]. ACCL are reputed by the frequency of late distant metastasis and local recurrence, though they are slow growing malignant tumors. Adenoid cystic carcinoma is categorized in three subtypes: Cribriform witch is the most common, tubular form with good prognosis and finally Solid subtype characterized by poor outcome [2-4]. We report, a new case of ACCL with unusual clinical presentation as we suspect a thyroid mass despite paraclinical investigations the initial site of the tumor couldn't be specified, it's only after the pathological examination of the operative specimen that the laryngeal origin was confirmed.

## Patient and observation

**Figure 1:**
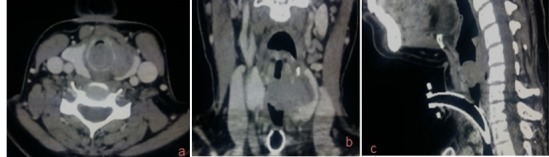
Contrast-enhaced cervical computed tomography: axial (A), coronal (B) and sagittal view (C): tumoral lesion between the left thyroid lobe, the left edge of the subglottic area of the larynx and the first tracheal ring filling all the lumen. The origin could be either the thyroÃ¯d gland or the larynx

**Figure 2:**
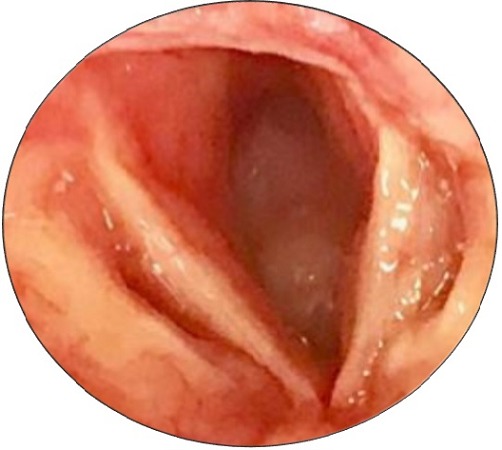
Endscopic view of the larynx showing a subglottic obstructive tumor which respects the arytenoids, the vocal folds and the hypopharynx

**Figure 3:**
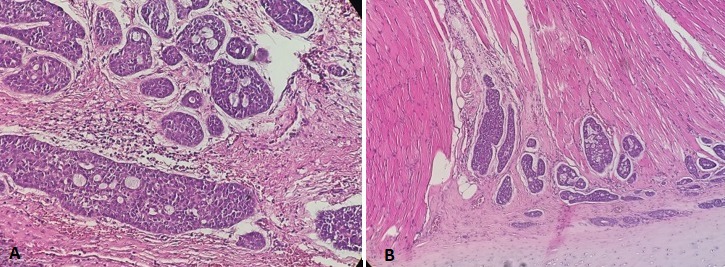
(A) cribriform growth pattern displaying several prominent pseudocysts surrounded by basaloid cells with hyperchromatic angulated nuclei (hematoxylin-eosin, x200); (B) low power view displaying the invasion of the muscle (hematowylin-eosin x100)

A 38-year-old woman with history of asthma, presented to our department with acute inspiratory dyspnea that required an emergency tracheotomy, without a history neither of hoarseness nor difficulty swallowing. Physical examination revealed a large anterior cervical mass without any lymphadenopathy. Cervical and Chest Computed Scan showed a tumor process between the thyroid lobe and the left edge of the subglottic area of the larynx and the first tracheal rings filling all the lumen. This process measures 34*25*50mm it respects the vocal cords and the arytenoids but invades the cricoid cartilage (Figure 1). The patient underwent panendoscopy under general anesthesia that shows the obstructive subglottic extension of the tumor with respect of the arytenoids and the hypopharynx (Figure 2). Multiple biopsies were done and confirmed the malignant salivary origin of the tumor. After multidisciplinary discussion the decision was to operate the patient. In fact, total laryngectomy and thyroidectomy with bilateral selective neck dissections (levels II- IV) was done. Macroscopic findings confirm the subglottic location of the tumor likewise anatomopathological examination showed an infiltrative carcinomatous proliferation, arranged in tubes and in cribriform massifs. These delineate glandular lights with mucoidal content (Figure 3). Overall, the morphological appearance of the tumor was compatible with Adenoid Cystic Carcinoma of the larynx that measures 3cm. The tumor was classified pT4aN0 as it infiltrates the thyroid, the cricoid cartilages and the thyroid gland. All limits (superior, inferior and posterior) were free from any tumor proliferation and both right and left lymphadenectomies were negatives. Adjuvant radiation therapy was indicated by the multidisciplinary team in this case.

## Discussion

Minor salivary gland tumors are rare, they represent less than 4% of all head and neck neoplasms [5-7 ]. Adenoid Cystic Carcinoma of minor salivary gland in larynx (ACCL) are extremely rare (<1%) [5]. The recent systematic review of Marchiano *et al*. reports that ACCL occurs in both sex with slightly predominance in men [5], the median and mean age reported is the fifties with clear predominance of white race [5,8]. We report a new case of Adenoid Cystic Carcinoma of Larynx occurred in non-Caucasian young woman aged 38 years old. The prognosis of ACCL depends deeply of tumor's characteristics, more than the lymph node extension and distant diseases. Indeed, lymph nodes are usually negative and distant metastasis rarely occurs, while the tumor size and invasion are often advanced at the diagnostic. The stage of the diagnosis is certainly stereotyped in our case as it was T4N0M0, nevertheless the clinical presentation was unusual. In fact, our patient presented a diagnosis of anterior cervical mass with acute dyspnea requiring an emergency tracheotomy. Clinical and paraclinical investigation didn't identified clearly if the starting point was thyroidian or laryngotracheal. In our knowledge, only 10 cases were reported in the literature with this unusual presentation [9]. In our case, the tumor was subglottic without invasion of neither vocal cords nor trachea. These findings are rare as the majority of cases reported in the literature had trachea as primary site [10-13]. Treatment modalities of ACCL remains non-standardized due to their rarity. Surgery is the cornerstone of treatment [8]; however, the appropriate surgical resection is not standardized yet. Indeed, some authors preferred radical surgery [1,3,14] even if wide local excision allowed to preserve laryngeal function. Their choice is argued by the relative radio resistance of this kind of tumor [15] as ACCL are radiosensitive but not radio curable [1,5]. However, British experience supports that combined treatment with preservative surgery and post-operative radiotherapy led to reduce loco-regional recurrences [16]. This objective was achieved in many studies with excellent loco-regional control thanks to addition of radiotherapy in various studies conducted in the MD Anderson Cancer Center [17,18]. In fact, some adverse features had significant influence on local control such as margins, tumor size, perineural invasion [18,19] that supported adjuvant local treatment independently of surgery's type. Moreover, the guidelines 2018 of National Comprehensive Cancer Network (NCCN) recommends, an adjuvant radiotherapy for adenoid cystic carcinoma of minor salivary gland even the surgery is complete and disregarding the adverse features (Category 2B) [20]. Adjuvant treatment's indications still controversial. Owing to the lack of randomized multicentric study, the personalized treatment should be the safe alternative taking in account the main adverse features such as close or positive margins, T3-4, intermediate or high grade neural and perineural spread, lymphatic or vascular invasion or lymph node metastases.

## Conclusion

Adenoid cystic carcinoma of the larynx is an extremely rare malignant neoplasm. Surgical resection with neck dissection is the main treatment of non-metastatic tumor, however combined modality treatments greatly improves loco-regional outcome.

## Competing interests

The authors declare no competing interests.

## References

[1] Testa D, Guerra G, Conzo G, Nunziata M, D'Errico G, Siano M (2013). Glottic-SubGlottic adenoid cystic carcinoma; a case report and review of the literature. BMC Surg.

[2] Marchiano E, Chin OY, Fang CH, Park RC, Baredes S, Eloy JA (2016). Laryngeal Adenoid Cystic Carcinoma: A Systematic Review. Otolaryngol Head Neck Surg.

[3] Zvrko E, Golubovic M (2009). Laryngeal adenoid cystic carcinoma. Acta Otorhinolaryngol Ital.

[4] Perzin KH, Gullane P, Clairmont AC (1978). Adenoid cystic carcinomas arising in salivary glands: a correlation of histologic features and clinical course. Cancer.

[5] Jones AV, Craig GT, Speight PM, Franklin CD (2008). The range and demographics of salivary gland tumours diagnosed in a UK population. Oral Oncol.

[6] Barnes L, Eveson JW, Reichart P (2005). World Health Organization, Classification of Tumours Pathology and Genetics of Head and & Neck Tumours.

[7] Wang X, Meng L, Hou T, Zheng C, Huang S (2015). Frequency and Distribution Pattern of Minor Salivary Gland Tumors in a Northeastern Chinese Population: A Retrospective Study of 485 Patients. J Oral Maxillofac Surg.

[8] Dubal PM, Svider PF, Folbe AJ, Lin HS, Park RC, Baredes S (2015). Laryngeal adenoid cystic carcinoma: a population-based perspective: laryngeal Adenoid Cystic Carcinoma. The Laryngoscope.

[9] Shirian S, Maghbool M, Aledavood A, Negahban S, Khademi B, Daneshbod Y (2017). Adenoid Cystic Carcinoma of the Larynx Presenting as a Thyroid Mass and Brief Literature Review. Acta Cytol.

[10] Yang PY, Liu MS, Chen CH, Lin CM, Tsao TCY (2005). Adenoid cystic carcinoma of the trachea: a report of seven cases and literature review. Chang Gung Med J.

[11] Idowu MO, Md CNP (2004). Adenoid Cystic Carcinoma: A Pitfall in Aspiration Cytology of the Thyroid. Am J Clin Pathol.

[12] Dixit R, Singhal A, Nuwal P (2010). Primary adenoid cystic carcinoma of trachea presenting as midline neck swelling and mimicking thyroid tumor: A case report and review of literature. Lung India.

[13] Na DG, Han MH, Kim KH, Chang KH, Yeon KM (1995). Primary adenoid cystic carcinoma of the cervical trachea mimicking thyroid tumor: CT evaluation. J Comput AssistTomogr.

[14] Liu W, Chen X (2015). Adenoid cystic carcinoma of the larynx: a report of six cases with review of the literature. Acta Otolaryngol (Stockh.).

[15] Saraydaroglu O, Coskun H, Kasap M (2011). Unique presentation of adenoid cystic carcinoma in postcricoid region: a case report and review of the literature. Head Neck Pathol.

[16] Avery CM, Moody AB, McKinna FE, Taylor J, Henk JM, Langdon JD (2000). Combined treatment of adenoid cystic carcinoma of the salivary glands. Int J Oral Maxillofac Surg.

[17] Fordice J, Kershaw C, El-Naggar A, Goepfert H (1999). Adenoid Cystic Carcinoma of the Head and Neck: Predictors of Morbidity and Mortality. Arch Otolaryngol. Neck Surg.

[18] Garden AS, Weber RS, Morrison WH, Ang KK, Peters LJ (1995). The influence of positive margins and nerve invasion in adenoid cystic carcinoma of the head and neck treated with surgery and radiation. Int J Radiat Oncol.

[19] Kokemueller H, Eckardt A, Brachvogel P, Hausamen JE (2004). Adenoid cystic carcinoma of the head and neck-a 20 years experience. Int J Oral Maxillofac Surg.

[20] Pfister DG (2017). NCCN Guidelines Index Table of Contents Discussion.

